# Disposal practices of long-lasting insecticidal nets, toxicity of the discarded nets and its potential implication on the development of pyrethroid resistance in *Anopheles gambiae* in Southern Ghana

**DOI:** 10.1186/s12936-026-05875-3

**Published:** 2026-03-26

**Authors:** Andreas A. Kudom, Leticia N. Anane, Stanley O. Okyere, Joana Ayettey, Jacob Nunoo, Ben A. Mensah

**Affiliations:** 1 Department of Conservation Biology and Entomology, University of Cape Coast, Cape Coast, Ghana; 2 Department of Applied Economics, University of Cape Coast, Cape Coast, Ghana

**Keywords:** *Anopheles gambiae*, Deltamethrin, Insecticide residue, Insecticide resistance, LLIN, PermaNet 3.0, Sublethal dose

## Abstract

**Background:**

Long-lasting insecticidal nets (LLINs) are central to malaria control, yet their management after loss of intended use remains poorly regulated in many African countries. Improper disposal or repurposing of LLINs may result in various environmental issues including release of insecticide residues and unintended exposure of malaria vectors. This study assessed the disposal practices in urban and rural households and evaluated the toxic effect of discarded LLINs against the malaria vector, *Anopheles gambiae*.

**Methods:**

We employed a mixed-methods approach comprising (i) a cross-sectional household survey to characterize LLIN ownership, use, and disposal practices in selected urban and rural communities in Ghana, and (ii) laboratory bioassays to evaluate the biological activity of insecticide residues from repurposed LLINs against field populations of *Anopheles gambiae.* Larval bioassays were conducted using water in which pieces of netting from LLIN had been soaked for short-term (1 day) and prolonged (7 days) exposure periods, while adult toxicity was assessed using cone bioassays. Susceptibility of the mosquito population to deltamethrin was also determined.

**Results:**

Repurposing of retired LLINs for agricultural fencing was common in both rural and urban settings (33%), alongside disposal by discarding in open environments. Water used to soak repurposed LLINs caused significant larval mortality and strong inhibition of pupation with survival probability of 15.1% after eight days, whereas adult exposure to the same nets resulted in negligible mortality.

**Conclusion:**

Repurposed LLINs retain biologically active insecticide residues capable of exerting strong sublethal effects on malaria vector larvae. Although this does not constitute direct evidence of resistance evolution, such exposure may contribute to selection pressures that maintain or amplify existing insecticide resistance. These findings highlight the need for clear national guidelines and community education on LLINs end-of-life management.

**Supplementary Information:**

The online version contains supplementary material available at 10.1186/s12936-026-05875-3.

## Introduction

Long-lasting insecticidal nets (LLINs) remain one of the most effective tools for malaria prevention [1]. Over 2 billion LLINs have been distributed globally [2] contributing substantially to reductions in malaria morbidity and mortality over the past two decades [3]. However, increasing attention has been drawn to the environmental and ecological implications of LLINs when they are no longer used for sleeping purposes. In many malaria-endemic countries, including Ghana, clear national policies or guidelines on the disposal of used LLINs are lacking. Consequently, households adopt a variety of disposal or repurpose practices, some of which may leave LLINs in prolonged contact with the environment [4].

Repurposing of LLINs for agricultural fencing, fishing, or domestic uses has been widely reported across Africa [5–7]. Unlike LLINs retained indoors, repurposed nets are typically exposed continuously to sunlight, rainfall, surface runoff, soil, and occasionally standing water. Such exposure may facilitate leaching of residual insecticides into surrounding environments [8–11], including potential mosquito breeding habitats. Pyrethroid insecticides, which constitute the active ingredient in most first-generation LLINs and remain a component of many second-generation nets [12], are known to degrade under sunlight [13, 14]. Indeed, pyrethroids have a degradation time of 50% below 60 days [13]. However, the octanol–water partition coefficient (K_ow_) values of some pyrethroids such as permethrin, cypermethrin and deltamethrin indicate their potential to persist in aquatic or terrestrial ecosystems at low concentrations [13]. Even sublethal concentrations of insecticides have been shown to affect insect development, survival, and tolerance to insecticides [15, 16].

**Fig. 1:**
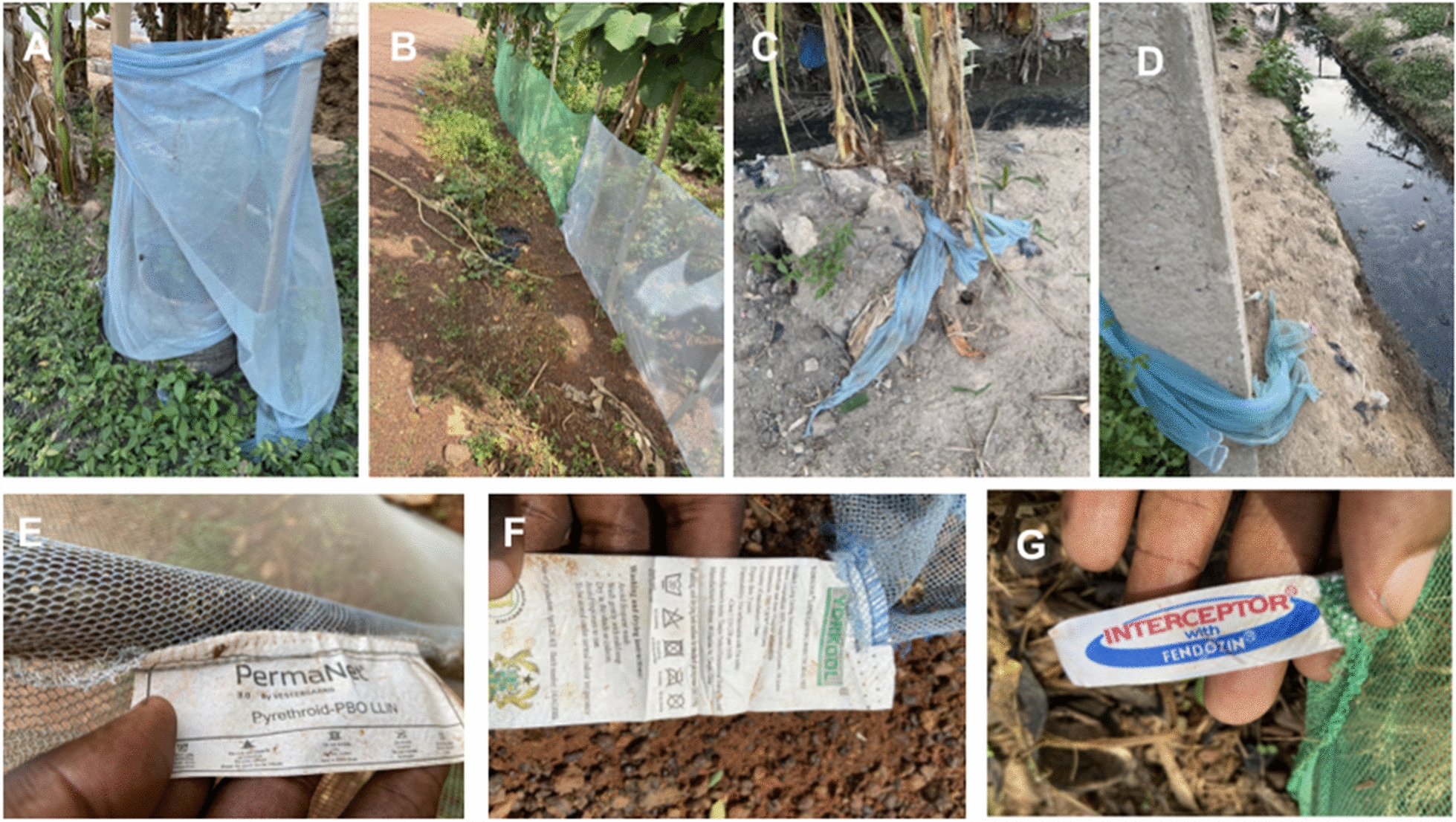
Different types of LLINs repurposed for fencing gardens or left in the open environment where they could be a potential shelter for adult mosquitoes or end up in mosquito breeding habitats; A-D - different repurposed LLINs; E-G- different brands of LLINs found in the environment.

This issue is of relevance in Ghana and other sub-Saharan African countries where malaria vectors such as *Anopheles gambiae* frequently breed in shallow, human-made water bodies near households and farms [17]. While previous studies have demonstrated that agricultural pesticide runoff can select for insecticide resistance in mosquito populations [18], empirical evidence linking LLIN disposal practices (Fig. 1) to biologically relevant exposure of malaria vectors remains limited.

We hypothesized that (i) retired LLINs are commonly repurposed or discarded in ways that leave them in open environments, (ii) such nets retain insecticide residues capable of exerting lethal or sublethal effects on *Anopheles gambiae*, a major malaria vector in Africa. To test these hypotheses, we combined household survey data with laboratory bioassays using field-collected mosquitoes.

## Materials and methods

### Study design and sites

**Fig. 2:**
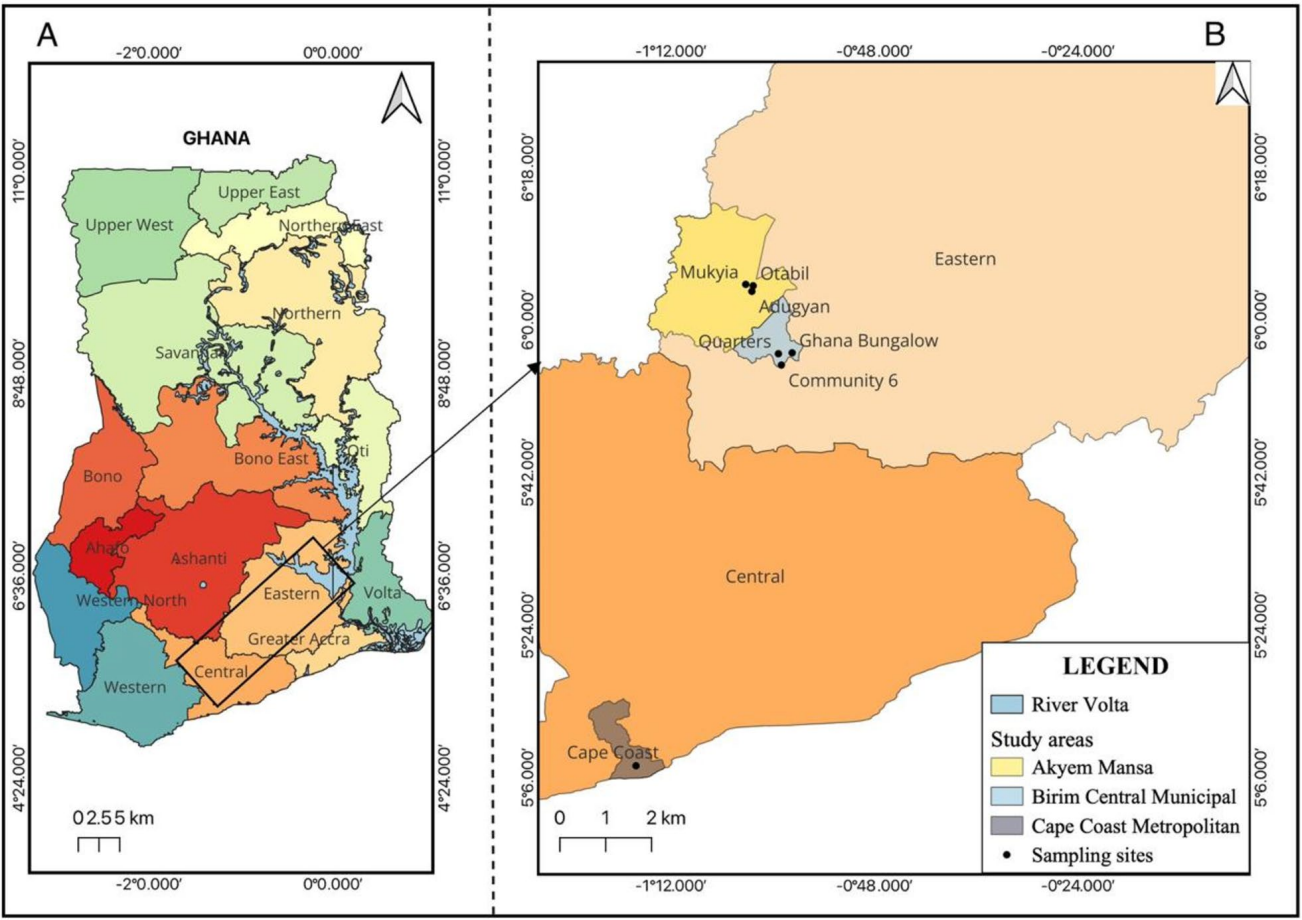
Map of Ghana (**A**) showing Cape Coast, Central region, Akim Oda and the rural communities (Mukyia, Adu-Gyan and Otabil), Eastern region (**B**) where the study was undertaken.

This study employed a mixed-methods design consisting of cross-sectional household and field surveys, and laboratory-based mosquito bioassays. The household survey was conducted in Akim Oda, an urban area and three rural communities (Mukyia, Otabil, and Adu-Gyan) in the Eastern Region of Ghana (Fig. 2). These communities were selected to represent contrasting urban and rural settings with high LLIN coverage and active agricultural practices, where repurposing of nets had been anecdotally observed. The field survey was also conducted in Akim Oda and Cape Coast to identify LLINs repurposed for fencing backyard gardens or farms. The laboratory bioassays were conducted at the Vector Biology and Control Laboratory, University of Cape Coast, using field populations of *Anopheles gambiae* collected in Cape Coast. The mosquito populations were collected as larvae from various sites in Cape Coast [17] and sent to the laboratory. Part of the larvae (3rd and 4th instar) were used for the larval bioassay, while other part was reared to adult and used for the adult bioassay.

### Household survey and sampling design

The primary sampling unit was the household, defined as individuals living together and sharing meals under the same roof. The household head or an informed adult member was interviewed per household. A systematic walk-through approach was used within each community, whereby households were approached consecutively along predefined routes until the target sample size was reached. Sample sizes for urban and rural settings were calculated a priori using Cochran’s formula, based on assumed disposal prevalence (70% for urban and 50% for rural) and a 95% confidence level [19]. A total sampling size of 522 were calculated for both study sites. In Akim Oda, the town was divided into four quadrants and equal number of households were surveyed (N = 138). In the rural areas, all the households in Adu-Gyan were targeted (N = 96), while equal numbers of households were surveyed in Mukyia and Otabil (N = 384).

During interviews, LLINs were explicitly defined as factory-treated mosquito nets, typically distributed through health facilities, mass distribution campaigns or bought from shops. Interviewers used locally familiar terms and, where necessary, visual aids to distinguish LLINs from untreated nets. To minimize reporting bias, interviewers employed neutral questioning, reassured respondents that there were no right or wrong answers and visually verified the presence and condition of nets where feasible.

The questionnaire sought to find out the following: if each household has ever used a LLIN before and if yes, whether it currently uses it; for households that currently use LLIN, the source of the net and whether somebody slept under the net the night before the interview. Furthermore, information was sought on whether a household had discontinued (retired) the use of a net before and if yes, how the net was discarded or disposed of.

In this study, “old” or “discarded” LLINs refer to nets that households reported as no longer suitable for sleeping due to wear, tearing, or perceived loss of effectiveness. The classification was based on respondents’ reports rather than verification of the manufacturer’s labelled lifespan or expiration date. Consequently, the term “discarded LLINs” in this study reflects user-defined retirement from routine use, rather than formally expired nets.

Consent to conduct the study was sought from the communal and individual level. Communal consent was obtained from the local or opinion leaders after the discussion about the study and request to conduct it in their communities. Individual consent was also obtained by discussing with each participant about the study and followed by a request to participate.

### Field survey of repurposed LLINs

A field survey was conducted in Akim Oda and Cape Coast to identify long‑lasting insecticidal nets (LLINs) repurposed for fencing backyard gardens or farms. The primary objective was to obtain field‑used nets for subsequent mosquito bioassays. LLINs repurposed for other agricultural activities, as well as nets from rural communities, were excluded to avoid potential contamination. Nets used for drying palm nuts were frequently contaminated with palm oil, while those used for covering seedlings were often contaminated with agricultural insecticides; such contaminants could influence mosquito mortality and confound bioassay results. In each town, a major street was randomly selected and systematically surveyed, including both private and public spaces. Nets with legible labels were documented, and information on insecticide type, manufacturing date, and release date was recorded. With owner consent, sections of selected nets were collected and stored for laboratory analysis.

### Laboratory bioassays

#### Larval bioassays

Five PermaNet 3.0 nets from the field survey were tested in the laboratory. For each net, two 11 cm by 11 cm pieces of netting were cut from each of the side net containing deltamethrin and the roof containing both deltamethrin and PBO. Similar pieces of netting were cut from new PermaNet 3.0 and untreated net, which served as a positive control and a negative control respectively.

Each piece (11 × 11 cm) of the LLINs was soaked in 250 ml of water for either 1 day (short-term exposure) or 7 days (prolonged exposure), representing plausible environmental contact durations such as rainfall events or standing water exposure. The soaking water was then used for larval bioassays. For each test, ten 3rd—4th instar larvae from the field were placed in a 250 ml plastic cup containing 50 ml of the water that was used to soak the net, and test for each piece of net was replicated five times. The larvae were monitored until they pupate or died.

#### Adult cone bioassays

Adult toxicity of the pieces of LLIN used for the larval bioassay was assessed using WHO cone bioassays. The bioassay was conducted following the WHO protocol (WHO/HTM/NTD/WHOPES/2013.1) with some modifications. WHO protocol recommends the exposure of the test mosquitoes to a 25 cm × 25 cm piece of netting for 3 min. However, in this study exposure time was extended to 30 min to maximise detection of residual toxicity in heavily aged nets; results are therefore interpreted as comparative toxicity assessments rather than standard WHO bio-efficacy estimates.

The test was performed with side and roof of each of the five-field collected PermaNet 3.0 LLIN, a new PermaNet 3.0 and untreated net. The new PermaNet 3.0 and untreated net were used as positive and negative controls, respectively. For each cone bioassay, five non-blood-fed, 2–5-day-old female *Anopheles* mosquitoes were used with five replications for each net. Knockdown and mortality were recorded at 1 h and 24 h post-exposure.

#### Insecticide susceptibility tests

Susceptibility of the mosquito population to deltamethrin was assessed using WHO diagnostic concentration (0.05%) and higher concentrations (0.25% and 0.5%) for adults, as well as larval dose–response assays to estimate lethal and inhibitory concentrations. The experiment consisted of a WHO test kit and treated papers, which were purchased from WHO (USM, Malaysia). A test with up to 25 three-to-five-day-old, non–blood-fed, female mosquitoes was exposed for 1 h to each of the insecticide-impregnated papers. The test was replicated three to five times for each insecticide concentration. A test with a paper treated with silicone oil was run in parallel to serve as a control. Final mortality was recorded 24 h after the exposure.

Deltamethrin solution purchased from WHO (USM, Malaysia) was used for the larval bioassay. Four concentrations were prepared by taking different volume of the stock solution (0.05%) and added to 200 ml of the water containing the larvae. Batches of 25 third or fourth-instar larvae were transferred to 250 ml plastic disposable cups containing 200 ml of water. Each concentration was replicated four times. Final mortality was recorded at 24 h after exposure or test was monitored until the death of the larvae or pupation for the pupal inhibition experiment. The pupae were removed from the experimental cup and placed in fresh water for emergence. The temperature in the test chamber was 28 °C and photoperiod of 12-h light followed by 12-h dark.

### Statistical analyses

Data were analysed using R software (version 4.2.3) [20]. Descriptive statistics were used to summarize the demographic characteristics of participants, as well as their responses on the ownership and use of LLINs, and disposal of LLINs respectively, using the gtsummary package (version 2.0.1) [20, 21]. Chi-square test was used to examine the association between the type of community (rural/urban) participants resided in and their demographic characteristics, as well as their responses on the ownership, use of LLINs, and their disposal respectively, using the gmodels package (version 2.19.1) [22]. However, Fisher’s exact test was employed when expected frequencies were less than 5 [23]. Similarly, t-test was also employed for questions that required responses in the form of continuous data (numbers) using the rstatix package (version 0.7.2) [24]. Log-linear analysis was also conducted to examine the relationships between demographic characteristics, disposal of LLINs, and type of community as well as participants’ awareness of any environmental concerns regarding disposal of LLINs, participants method of disposing off used LLINs respectively using the gmodels package (version 2.19.1). The larval bioassay data was analysed using the BioRssay package (version 1.1.0) [25] while the data from the cone bioassay were visualized using the ggplot2 package (version 3.5.1) [26]. However, larval survival data were analysed using survival analysis methods, with pupation treated as censored observations using ggsurvfit package (version 1.1.0) [27]. Control mortality was monitored to account for natural background mortality.

## Results

### Household survey

#### Demographic characteristics

A total of 520 households were successfully interviewed in both the rural and urban communities. The demographic characteristics of the participants have been summarised in Table 1. The educational background of the participants differed between the rural and urban areas (χ^2^ = 144.676, df = 3, *p* =  < 0.001). While most of the participants from the rural areas had non-formal (38%, N = 385) or had primary education (49%, N = 385), most of the participants in the urban area had secondary (34%, N = 134) or tertiary education (33%, N = 134). There was a significant difference in the occupation of the participants between the rural and urban communities (χ^2^ = 51.997, d.f. = 2, p =  < 0.001). The major occupation of the participants in the rural areas was self-employed, which was either farming or trading, whereas most of the participants in the urban areas were trading (self-employed) or government workers (Table 1).

**Table 1:** Demographic characteristics of participants in the survey in urban (Akim Oda) and rural (Akyemansa Districts) communities in the Eastern Region of Ghana

Variable	Category	Community		Total
		Rural	Urban	
Sex	Male	187 (48.0%)	67 (50.0%)	254 (49.0%)
	Female	199 (52.3%)	67 (50.0%)	266 (51.0%)
	Total	386 (100.0%)	134 (100.0%)	520 (100.0%)
Age	18–40 years	51 (13.2%)	31 (23.0%)	82 (15.9%)
	41–59 years	333 (86.5%)	101 (77.0%)	434 (83.9%)
	> 60 years	1 (0.3%)	0 (0.0%)	1 (0.2%)
	Total	385 (100.0%)	132 (100.0%)	517 (100.0%)
Education	Non-formal	146 (37.8%)	15 (11.0%)	161 (31.0%)
	Primary	189 (48.9%)	30 (22.0%)	219 (42.0%)
	Secondary	29 (7.5%)	45 (34.0%)	74 (14.0%)
	Tertiary	22 (5.7%)	44 (33.0%)	66 (13.0%)
	Total	386 (100.0%)	134 (100.0%)	520 (100.0%)
Occupation	Government worker	23 (6.0%)	37 (28.0%)	60 (12.0%)
	Self-employed	314 (81.0%)	72 (54.0%)	387 (74.0%)
	Not employed	49 (13.0%)	24 (18.0%)	73 (14.0%)
	Total	386 (100.0%)	134 (100.0%)	520 (100.0%)

#### Ownership, use and disposal of LLIN

Most households reported prior use of LLINs (99%), with high current usage at the time of the survey (87%). However, LLINs usage differed between the urban and the rural areas (χ^2^ = 3.298, d.f. = 1, *p* = 0.069). Most of the participants (64%) using LLIN stated that they obtained the LLINs from hospitals, while 16% stated that they obtained theirs through mass distribution (S1). Half of the participants (50%) stated that they obtained the LLINs they were using between one to three years ago, while 35% stated that they obtained their net less than a year ago. Generally, about 79% of the participants stated that somebody slept under the net in the night before the interview and there was no significant difference in the urban and the rural area (χ^2^ = 0.302 d.f. = 1, *p* = 0.582). In contrast, there was a significant difference among the participants in urban and rural areas on their responses on who slept under the LLIN in their household the night before the interview (Fisher’s exact test = 109.988, *p* =  < 0.001). Interestingly, most of the people who slept under the net in the rural communities were both parents and children (67%), but children were the majority that slept under the net in urban communities (55%) (S1).

More than half of the participants (57%) stated that the first time they used LLIN was within the last five years. Most of the participants (80%) had discontinued the use of at least one LLIN before. However, 72.5% had disposed of one to three nets since they started using LLIN. The disposal practices of the participants have been summarised in S1 (supplementary data).

Overall, 33% reported repurposing retired LLINs for fencing backyard gardens or farms, 22% used them for domestic activities such as screening windows or trap doors, while 21% disposed of them in refuse dumps. The statistical test (χ^2^ = 91.331, d.f. = 2, p < 0.001) indicates a highly significant difference in how retired LLINs are repurposed between rural and urban communities. For example, only 25% repurposed the retired LLINs for fencing backyard garden/farm in rural areas whereas 61% used them for the same purpose in urban areas. Also, 25% of the participants in the rural areas repurposed the retired LLINs for other agricultural activities such as drying palm nut fruits or covering rice or cocoa seedlings whereas there were no such activities in the urban areas.

Majority of the participants (75%) stated that they were not aware of any environmental concerns about the reused of the old LLINs in agricultural activities, while 25% stated otherwise. Among those who stated that they were aware of environmental concerns about the repurposing of LLIN into agricultural activities, a higher proportion (77%) stated that LLINs influence their crops, while 22% stated that the old LLIN affects the fertility of the soil.

A three-way log-linear analysis was conducted to determine if there is a relationship between community type (rural/urban), demographics of the participants, and LLIN disposal practices of the participants. However, there was no significant relationship between the LLIN disposal practices of the participants and the sex of participants (χ^2^ = 3.909, *p* = 0.689), age of the participants (χ^2^ = 7.782, *p* = 0.802), educational status of the participants (χ^2^ = 7.072, *p* = 0.99), and occupation of participants (χ^2^ = 18.819, *p* = 0.093) respectively across both communities. Similarly, there was no significant relationship between participant’s awareness of any environmental concerns about disposal of LLINs and their LLIN disposal practices across both communities (χ^2^ = 1.224, *p* = 0.976).

#### Information on the LLINs that were being used as fencing materials in Akim Oda and Cape Coast

**Fig. 3:**
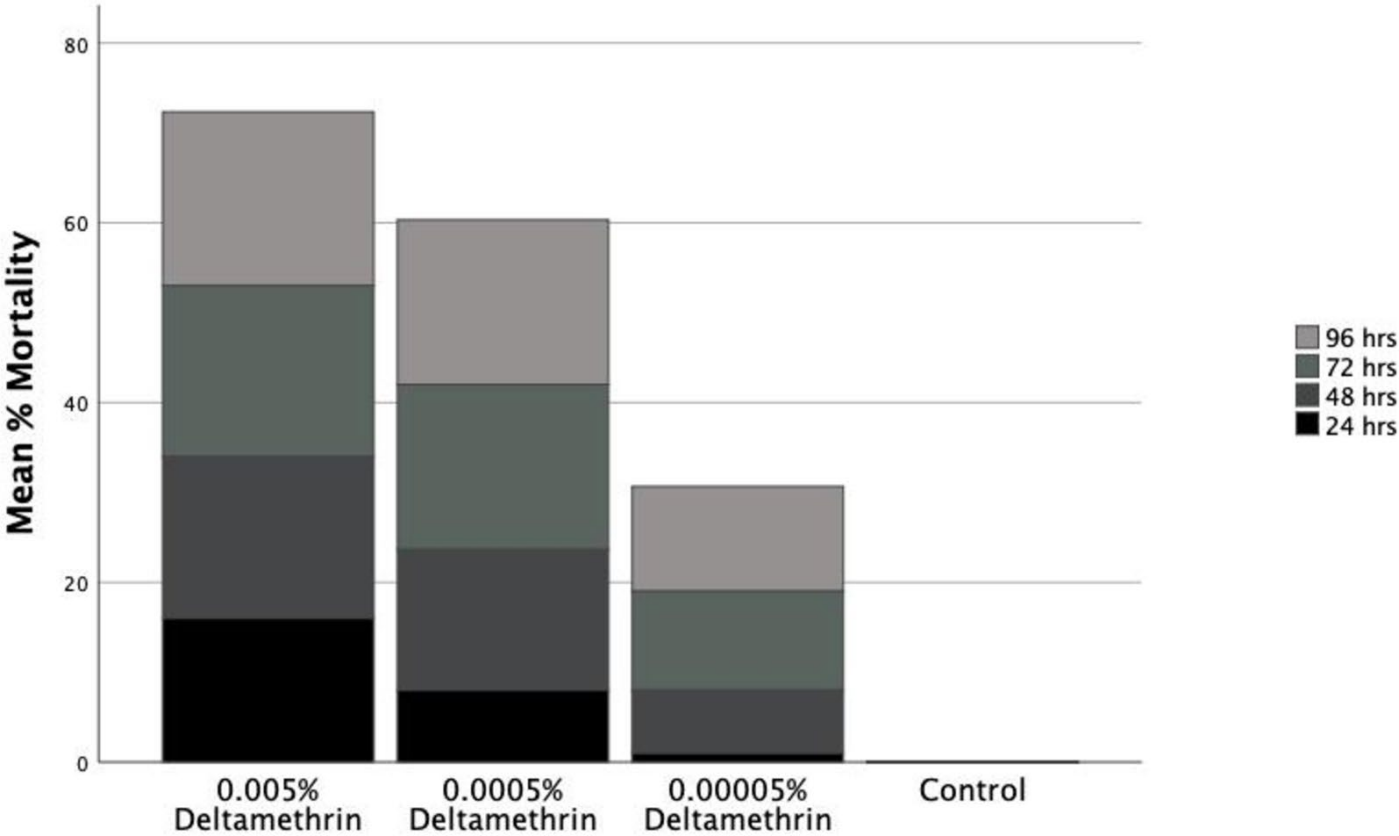
Mean %mortality of larvae of *An. gambiae* s.l exposed to different concentrations of deltamethrin for 96 h

Most of the nets in the field had the information label missing or faded. However, 18 nets that had information on them had a median (IQR) age of 5 years (3.75) based on the released date. About 89% were used for fencing backyard gardens (Fig. 3). Only one of the nets was treated with permethrin, while 44% were treated with alpha cypermethrin and 50% were treated with deltamethrin. Among the nets treated with deltamethrin, 44.4% belonged to the second generation LLINs that had PBO synergist incorporated into the roof of the net.

### Laboratory bioassay

#### Toxicity of LLIN residues to mosquito larvae

From the larval bioassay, LC_50_ (CI) of deltamethrin against the field population was 0.0008% (< 0.0001–0.0298). However, the IE_50_ (CI) was 0.0001% (< 0.0001- 0.0135). The insecticide still had an enormous effect after the 24 h exposure (Fig. 3). For instance, concentration of 0.00005% of deltamethrin caused less than 10% mortality after 24 h but inhibited about 60% of the larvae from pupating.

**Fig. 4:**
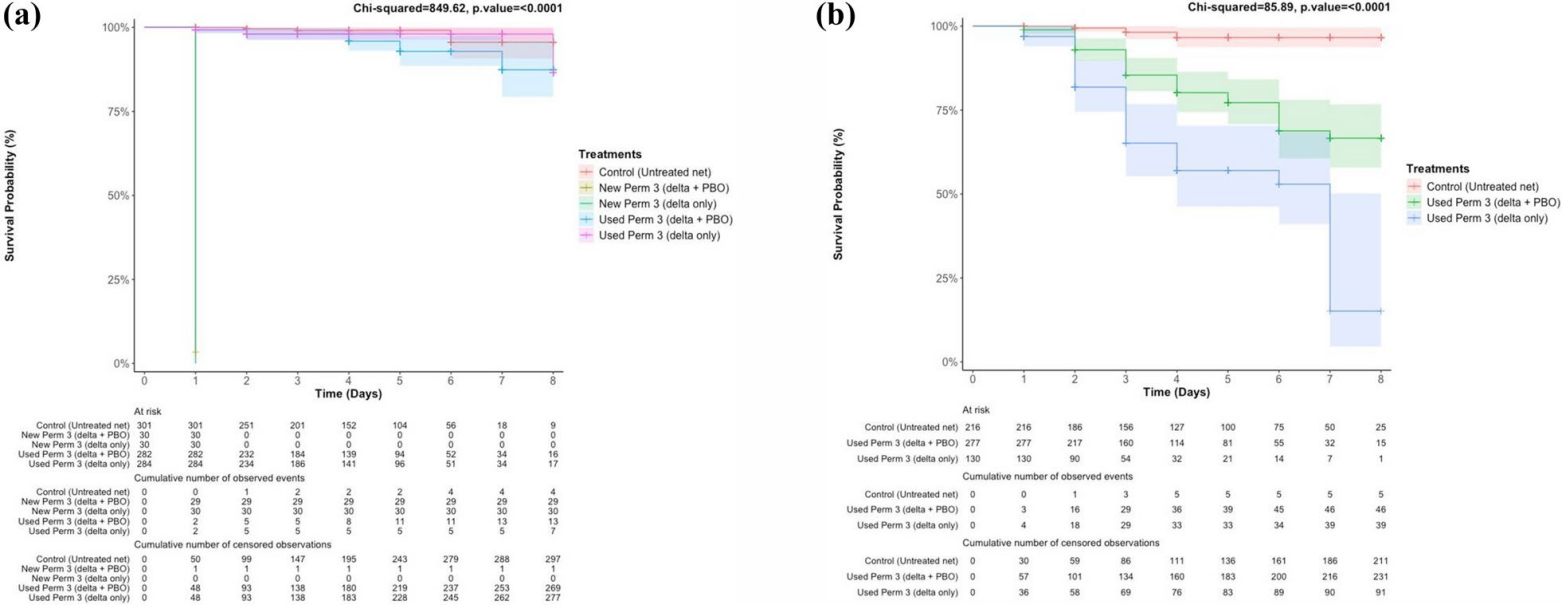
**a, b** Kaplan–Meier survival curves (lines) with 95% confidence intervals (coloured bands) of mosquito larvae reared in water **a** from new and used Perm Net 3 soaked for 1 day, **b** used Perm Net 3 soaked for 7 days. Survival probabilities of wild populations of *Anopheles gambiae* s.l. were compared under laboratory conditions; + indicates pupation that occurred before the end of the experiment and was treated as censored data

Larval exposure to water used to soak repurposed LLINs resulted in significantly reduced survival and strong inhibition of pupation, particularly after 7 days of soaking (Fig. 4). Survival probability declined steadily over time, dropping to low levels in treatments involving prolonged soaking of used LLINs; 15.1% versus 87% survival probability by day 8 of larvae exposed to the used LLINs that were soaked for 7 days and 1 day respectively, while survival probability remained high in negative controls (> 95%). Low mortality observed in untreated controls was consistent with expected background mortality under laboratory conditions. The roof and side of the new LLIN, respectively caused 97% and 100% mortality of the mosquito larvae within 24 h.

#### Toxicity of LLIN residues to adult mosquitoes

**Fig. 5:**
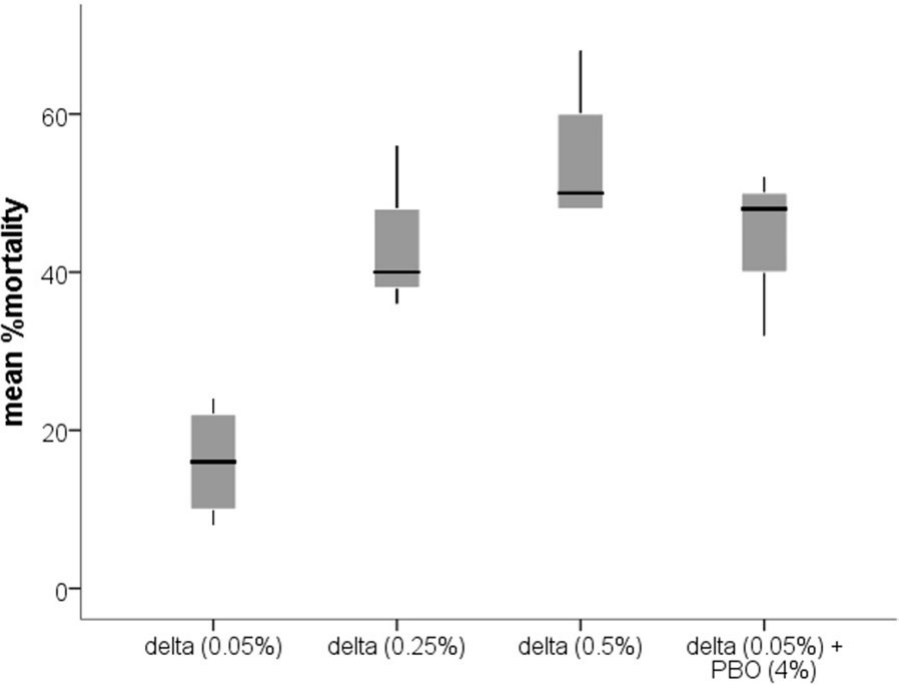
Mean % mortality of adult *Anopheles gambiae* s.l. against different concentrations of deltamethrin insecticide from WHO susceptibility bioassay

**Fig. 6:**
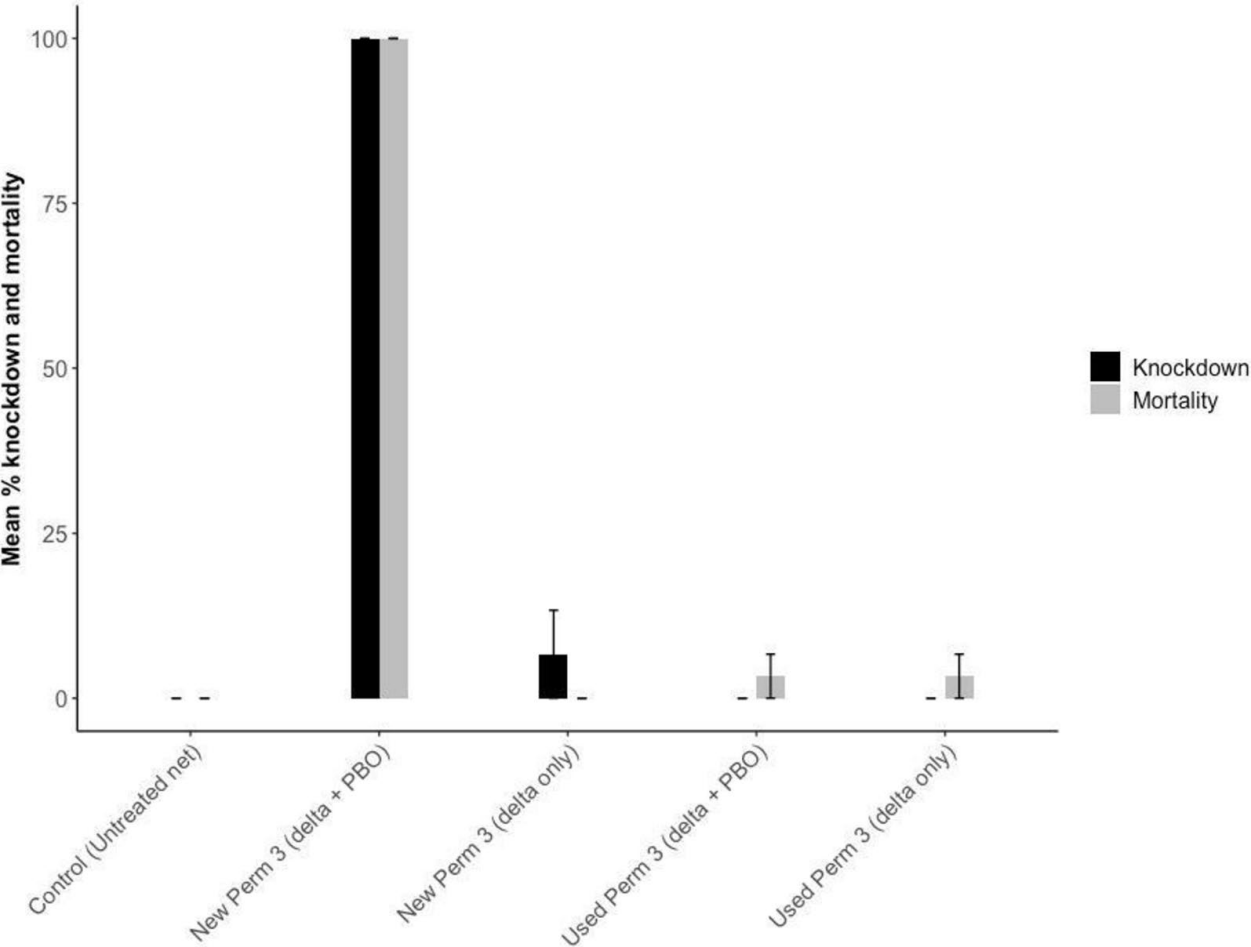
The mean percentage knockdown and mortality of *Anopheles gambiae* against new and used LLIN from cone bioassay

The field populations of *Anopheles gambiae* exhibited high resistance to deltamethrin (Fig. 5). The diagnostic concentration (0.05%) caused less than 16% mortality whereas 10 folds of the diagnostic concentration (0.5%) caused about 50% mortality. Interestingly, the diagnostic concentration plus PBO (4%) increased the mortality from around 16% to about 50%. From the cone test, exposure to the used LLIN resulted in negligible adult mortality, whereas new PBO-containing LLIN roof panels caused complete knockdown and mortality (Fig. 6).

## Discussion

This study found high ownership and use of insecticide-treated nets (ITNs) among participants, likely attributable to the free distribution of long-lasting insecticidal nets (LLINs) by Ghana’s National Malaria Elimination Programme (NMEP). The NMEP conducts mass LLIN distribution campaigns every three years, complemented by continuous distribution through antenatal and child welfare clinics. However, patterns of LLINs usage differed between rural and urban settings. In rural areas, both parents and children commonly slept under LLINs at night preceding the survey, whereas in urban areas, use was predominantly reported among children. This finding is consistent with previous studies reporting higher LLIN utilization in rural households compared with urban households in Ghana [28, 29], likely reflecting the higher malaria burden and stronger programmatic focus in rural areas [30].

Over half of respondents reported initiating LLIN use within the past five years, likely reflecting recent scale-up of mass and continuous distribution campaigns as well as recall limitations. Retired LLINs were commonly repurposed for agricultural fencing, while a substantial proportion were discarded in open environments. This observation is consistent with findings from Kenya, where widespread reuse of LLINs for farming has been reported [31]. In contrast, studies from other regions of Ghana have documented lower levels of reuse, with disposal through burning or dumping being more common [4]. The predominance of agricultural livelihoods in the present study communities likely explains the high level of LLIN reuse. Nevertheless, improper disposal practices such as open burning or dumping were also observed. Burning LLINs may release toxic compounds, including dioxins, which may pose potential risks to human health [9].

It is important to note that retirement of LLINs in household settings is often determined by perceived deterioration or physical damage rather than the manufacturer-specified lifespan, which may influence how communities decide to repurpose or dispose of nets. The World Health Organization recommends the disposal of LLINs through high-temperature incineration or, where this is unavailable, burial in non-permeable soil away from water sources [11]. Although this study did not assess participants’ awareness of recommended disposal practices, evidence from other settings suggests limited knowledge among both community members and key stakeholders [32, 33]. Strengthening health education on appropriate LLIN disposal is therefore essential to promote environmentally safe practices and reduce environmental contamination.

Field observations indicated that most LLINs repurposed as fencing materials were second-generation LLINs possessing both pyrethroid insecticides and piperonyl butoxide (PBO). Cone bioassay results showed minimal mortality of adult mosquitoes following exposure to the sides and roofs of the used LLINs, as well as the sides of new LLINs, reflecting the high level of deltamethrin resistance in the local mosquito population. In contrast, exposure to the roof of new LLINs, which contains both deltamethrin and PBO, resulted in complete mosquito mortality. This finding supports existing evidence that PBO-treated LLINs are more effective against pyrethroid-resistant vectors by inhibiting metabolic resistance mechanisms [34, 35]. Notably, the exposure time used in this study exceeded the standard WHO diagnostic exposure duration.

This study demonstrates that repurposed LLINs, particularly those used for agricultural fencing, retain biologically active insecticide residues capable of exerting strong sublethal effects on malaria vector larvae. Current insecticide evaluation methods primarily focus on immediate mortality and often overlook delayed or sublethal effects. In this study, the concentration of deltamethrin required to inhibit pupation in 50% of larvae was substantially lower than the concentration required to cause equivalent mortality within 24 h. These findings suggest that sublethal concentrations, while causing minimal short-term mortality, can significantly disrupt larval development and may contribute to the selection and persistence of resistant mosquito populations. For example, Ablode et al. [15] provided evidence of the development of deltamethrin resistance in an *Aedes aegypti* population after sublethal exposure to pyrethroid‑based mosquito coils for 16 generations.

The pronounced larval effects observed, contrasted with the minimal adult mortality recorded in resistant mosquito populations, highlight a potential disconnect between standard adult bioassay endpoints and ecologically relevant selection pressures acting during immature stages. Although repurposed LLINs represent a localized rather than ubiquitous exposure pathway, they provide a plausible mechanism by which low-level insecticide exposure may contribute to the maintenance or amplification of resistance in specific micro-environments.

Discarded nets in open environments may represent an additional exposure pathway, suggesting that multiple disposal practices could contribute to environmental contamination. It has already been established that residual insecticides from LLINs can be toxic to aquatic organisms, particularly certain fish species [11]. Effects such as decreased egg fertilization rates and overall declines in fish productivity have been reported in several studies [6, 18, 36]. We acknowledge that chemical quantification of insecticide residues was beyond the scope of this study; however, the biological responses observed provide functional evidence of residual insecticidal activity. The household survey captured self-reported disposal and repurposing practices for retired LLINs. The study did not include systematic observation or enumeration of repurposed nets within households or surrounding environments, and therefore the prevalence of households currently using repurposed nets could not be directly determined. Consequently, reported frequencies reflect respondents stated practices rather than direct measurements of household-level prevalence. Future studies incorporating environmental observation or structured household inspections would help quantify the true prevalence and spatial distribution of repurposed LLINs within communities.

## Conclusion

LLINs remain an essential tool for malaria prevention. However, our findings indicate that repurposed LLINs left in open environments retain insecticide residues capable of significantly affecting the survival and development of malaria vector larvae. Although there is no direct evidence of resistance evolution, such exposures may contribute to selection pressures that sustain existing insecticide resistance. Clear national policies and strengthened community education on LLIN end-of-life management are therefore essential components of sustainable malaria control strategies.

## Supplementary Information

Supplementary Material 1. S1 Participants’ responses on the ownership, use and disposal of LLINs in urbanand ruralcommunities in the Eastern Region of Ghana

## Data Availability

All data generated or analysed during this study are included in this manuscript and its supplementary information file.
