# Supplementary material for: Disposal practices of long-lasting insecticidal nets, toxicity of the discarded nets and its potential implication on the development of pyrethroid resistance in Anopheles gambiae in Southern Ghana
Source: Malar J. 2026 Mar 26;25:193. doi: 10.1186/s12936-026-05875-3 (PMC13141371; doi:10.1186/s12936-026-05875-3)
Supplement: Supplementary file 1 — Supplementary Material 1. S1 Participants’ responses on the ownership, use and disposal of LLINs in urbanand ruralcommunities in the Eastern Region of Ghana [file 12936_2026_5875_MOESM1_ESM.docx]

S1: Participants’ responses on the ownership, use and disposal of LLINs in urban (Akim Oda) and rural (Akyemansa Districts) communities in the Eastern Region of Ghana

| **Question** | **Response** | **Community** | |  |
| --- | --- | --- | --- | --- |
|  |  | **Rural** | **Urban** | **Total** |
| Have you ever used a LLIN before | Yes | 382 (99.0%) | 134 (100.0%) | 516 (99.0%) |
|  | No | 3 (0.8%) | 0 (0.0%) | 3 (0.6%) |
|  | Total | 385 (100.0%) | 134 (100.0%) | 519 (100.0%) |
| Do you currently have LLIN in your house? | Yes | 339 (88.0%) | 110 (82.0%) | 449 (87.0%) |
|  | No | 45 (12.0%) | 24 (18.0%) | 69 (13.0%) |
|  | Total | 384 (100.0%) | 134 (100.0%) | 518 (100.0%) |
| Where did you obtain the LLIN from? | Mass distribution | 83 (37.8%) | 1 (11.0%) | 84 (16.0%) |
|  | Antenatal | 25 (6.6%) | 8 (6.0%) | 33 (6.4%) |
|  | Pharmacy shop | 5 (1.3%) | 11 (8.3%) | 16 (3.1%) |
|  | Hospital | 240 (63.0%) | 90 (68.0%) | 330 (64.0%) |
|  | None | 28 (7.3%) | 23 (17%) | 51 (9.9%) |
|  | Total | 381 (100.0%) | 133 (100.0%) | 514 (100.0%) |
| When did you get the current LLIN? | More than 3 years | 11 (2.9%) | 1 (0.7%) | 12 (2.3%) |
|  | 1-3 years | 151 (40.0%) | 105 (78.0%) | 256 (50.0%) |
|  | Less than 1 year | 175 (46.0%) | 4 (3.0%) | 179 (35.0%) |
|  | None | 40 (11.0%) | 24 (18.0%) | 64 (13.0%) |
|  | Total | 377 (100.0%) | 134 (100.0%) | 511 (100.0%) |
| Did anybody in your household sleep under a LLIN last night? | Yes | 272 (78.0%) | 107 (80.0%) | 379 (79.0%) |
|  | No | 76 (22.0%) | 26 (20.0%) | 102 (21.0%) |
|  |  |  |  |  |
|  | Total | 348 (100.0%) | 133 (100.0%) | 481 (100.0%) |
| Who slept under the LLIN in your household last night? | Parent | 8 (2.2%) | 2 (1.5%) | 10 (2.0%) |
|  | Children | 40 (11.0%) | 73 (55.0%) | 113 (22.0%) |
|  | Parent & Children | 248 (67.0%) | 32 (24.0%) | 280 (56.0%) |
|  | None | 74 (20.0%) | 26 (20.0%) | 100 (20.0%) |
|  | Total | 370 (100.0%) | 133 (100.0%) | 503 (100.0%) |
| When was the first time you used LLIN? | More than 10 years ago | 19 (5.0%) | 0 (0.0%) | 19 (3.7%) |
|  | 5-10 years | 103 (27.0%) | 46 (34.0%) | 149 (29.0%) |
|  | 1-5 years | 204 (54.0%) | 88 (66.0%) | 292 (57.0%) |
|  | Less than a year | 51 (14.0%) | 0 (0.0%) | 51 (10.0%) |
|  | Total | 377 (100.0%) | 134 (100.0%) | 511 (100.0%) |
| How many LLINs have you disposed since you started using it? | 0 | 44 (12.2%) | 39 (29.1%) | 83 (16.8%) |
|  | 1-3 | 274 (75.9%) | 85 (63.4%) | 359 (72.5%) |
|  | 4 or more | 43 (11.9%) | 10 (7.5%) | 53 (10.7%) |
|  | Total | 361 (100.0%) | 134 (100.0%) | 495 (100.0%) |
| What do you do with LLIN that you have discontinued using? | Burn it | 0 (0.0%) | 6 (6.3%) | 6 (1.5%) |
|  | Domestic activities | 22 (6.9%) | 0 (0.0%) | 22 (5.3%) |
|  | fencing backyard garden/farm | 77 (24.0%) | 58 (61.0%) | 135 (33.0%) |
|  | Give it out | 69 (22.0%) | 7 (7.4%) | 76 (18.0%) |
|  | Keep it | 6 (1.9%) | 0 (0.0%) | 6 (1.5%) |
|  | Other agricultural activities | 79 (25.0%) | 0 (0.0%) | 79 (19.0%) |
|  | Throw them away | 64 (20.0%) | 24 (25.0%) | 88 (21.0%) |
|  | Total | 317 (100.0%) | 95 (100.0%) | 412 (100.0%) |
| Are you aware of any environmental concerns about the disposal of LLINs? | Yes | 75 (20.0%) | 53 (40.0%) | 128 (25.0%) |
|  | No | 300 (80.0%) | 80 (60.0%) | 380 (75.0%) |
|  | Total | 375 (100.0%) | 133 (100.0%) | 508 (100.0%) |
| If yes give some of the concerns | Affects the fertility of soil and growth of plants | 1 (1.4%) | 0 (0.0%) | 1 (0.8%) |
|  | Affect the fertility of the soil | 14 (20.0%) | 13 (25.0%) | 27 (22.0%) |
|  | Affect the growth of plants | 56 (79.0%) | 39 (75.0%) | 95 (77.0%) |
|  | Total | 71 (100.0%) | 52 (100.0%) | 123 (100.0%) |
